# Real-time imaging of structure and dynamics of transmembrane biomolecules by FRET-induced single-molecule fluorescence attenuation

**DOI:** 10.52601/bpr.2021.210030

**Published:** 2021-12-31

**Authors:** Dongfei Ma, Wenqing Hou, Chenguang Yang, Shuxin Hu, Weijing Han, Ying Lu

**Affiliations:** 1 Songshan Lake Materials Laboratory, Dongguan, Guangdong 523808, China; 2 Beijing National Laboratory for Condensed Matter Physics, Institute of Physics, Chinese Academy of Sciences, Beijing 100190, China; 3 University of Chinese Academy of Sciences, Beijing 100049, China

**Keywords:** Single-molecule fluorescence imaging, Dynamics of membrane proteins

## Abstract

Tracking the transmembrane topology and conformational dynamics of membrane proteins is key to understand their functions. It is however challenging to monitor position changes of individual proteins in cell membranes with high sensitivity and high resolution. We review on three single-molecule fluorescence imaging methods — SIFA, LipoFRET and QueenFRET — recently developed in our lab for studying the dynamics of membrane proteins. They can be applied, progressively, to investigate membrane proteins in solid-supported lipid bilayers, artificial liposome membranes and live-cell plasma membranes. The techniques take advantage of the energy transfer from a fluorophore to a cloud of quenchers and are able to extract in real time positions and position changes of a single fluorophore-labeled protein in the direction normal to the membrane surface. The methods have sub-nanometer precision and have proved powerful to investigate biomolecules interacting with bio-membranes.

## INTRODUCTION

Plasma membranes play important roles in many metabolic processes. They control the information flow and substance trafficking inward or outward the cells and serve as the stage for many biochemical reactions (Almen* et al.* 2009; Bretscher and Raff 1975). The functions of plasma membranes rely on membrane-based biomolecules, especially membrane proteins. Involved in processes such as ligand–receptor interactions (Latorraca* et al.* 2017), transmembrane transport (Bennett* et al.* 2019; de Lera Ruiz and Kraus 2015) and lipid organization (Lingwood and Simons 2010; Sonnino and Prinetti 2013), these proteins undergo multiple conformational changes upon activation and are precisely regulated (Contreras* et al.* 2010; Latorraca* et al.* 2017; Leth-Larsen* et al.* 2010; Nishida* et al.* 2014). Therefore, probing the dynamics of membrane-bound proteins as well as the interaction between the proteins and the membranes are crucial for understanding their functions and the underlying mechanisms. Over years, structural analysis techniques such as X-ray crystallography (Andersson* et al.* 2019; Lieberman* et al.* 2013), nuclear magnetic resonance (Bibow and Hiller 2019; Nishida* et al.* 2014; Radic and Pattanaik 2018), neuron reflectivity (Hellstrand* et al.* 2013), and the fast-developing electron microscopy techniques (Cheng 2018; Garcia-Nafria and Tate 2020; Yao* et al.* 2020) have provided valuable information about the structures of membrane-interacting macromolecules. However, these methods either acquire only static conformations, or work with ensembles of molecules. Because the macromolecular machines often work in the timescale below seconds and may switch among multiple conformational states (Haberl* et al.* 2009; Prakash and Gorfe 2019; Volkman* et al.* 2001), the time- or ensemble-averaged results could not fully uncover the mechanism of membrane proteins. Single-molecule methods are able to explore the molecular details of various physiological processes in real time, including immune responses, membrane fusion and fission, phase separation and lipid raft formation.

Single-molecule manipulation methods have long been applied in membrane protein studies. Taking a good example, the atomic force microscopy (AFM) has ultra-high axial resolution and the capability to exert forces on the molecules understudy. AFM is good for detecting localization and folding/unfolding of membrane proteins (Jefferson* et al.* 2018; Muller* et al.* 2006). It was used to characterize the assembly of FoF1-ATP synthases (Stahlberg* et al.* 2001) and the folding of β-barrel proteins (Thoma* et al.* 2018), revealing the relationship between the protein’s mechanical properties and functions. Another example is the patch clamp, which has long been used to monitor the activation and regulation of ionic channels (Bebarova 2012).

Single-molecule fluorescence is a good choice for membrane-protein studies. Fluorescence resonance energy transfer (also called Förster resonance energy transfer, FRET), which occurs on the occasion that one fluorophore’s emission spectrum has overlapped with another’s absorption, is sensitive to sub-nanometer changes of distances between two fluorophores (Ha* et al.* 1996). It has been widely used to probe the folding of membrane proteins, intra-molecular movements of domains, and inter-molecular movements of interacting proteins (Krainer* et al.* 2019). It can also serve as indicators of multiple membrane-associated events (Diao* et al.* 2010, 2011; Fitzgerald* et al.* 2019). Examples include the plug movement during initiation of translocation of SecYEG (Fessl* et al.* 2018), the folding kinetics of the helical protein Mistic (Krainer* et al.* 2018), the ligand interactions and dimerization of GPCRs (Asher* et al.* 2021), and the content mixing during the SNARE-mediated membrane fusion (Diao* et al.* 2011). Combined with the patch clamp, single-molecule FRET was adopted to explore the conformational changes of ion channels, such as the gramicidin ion channel (Harms* et al.* 2003) and the NMDA receptor ion channel (Sasmal and Lu 2014).

The relative position changes between protein domains probed by single-molecule FRET provide crucial information about the mechanism of membrane proteins. Under some conditions, in order to explore the orientation and the insertion of proteins in the membranes, the movements of a single domain relative to the membrane are as informative and fundamental as the relative movements of the domains. However, the lateral fluidity and fluctuations of lipid bilayers pose obstacles to the detection of the movements of membrane proteins. In addition, to extract the vertical component of the protein’s movements with respective to the membrane, the sub-nanometer resolution is required because the thickness of plasma membranes is in the nanometer scale (Goksu* et al.* 2009; Morandat* et al.* 2013).

Here, we review on three novel fluorescence imaging techniques developed recently in our lab for studying the membrane–protein interactions and the dynamics of macromolecules in the plasma membranes. They are the surface-induced fluorescence attenuation (SIFA) (Li* et al.* 2016; Ma* et al.* 2018) which uses supported lipid bilayers, FRET with quenchers in liposomes (LipoFRET) (Ma* et al.* 2019a) which is based on liposomes, and FRET with quenchers in the extracellular environment (QueenFRET) (Hou* et al.* 2021) which works on live-cell membranes. All the three methods can monitor the movements of a labeled site of the membrane proteins (or other bio-molecules) in the direction normal to the membrane surface in real time, with sub-nanometer precision.

## SURFACE-INDUCED FLUORESCENCE ATTENUATION (SIFA)

### The principle and experiment setup of SIFA

**Figure 1:**
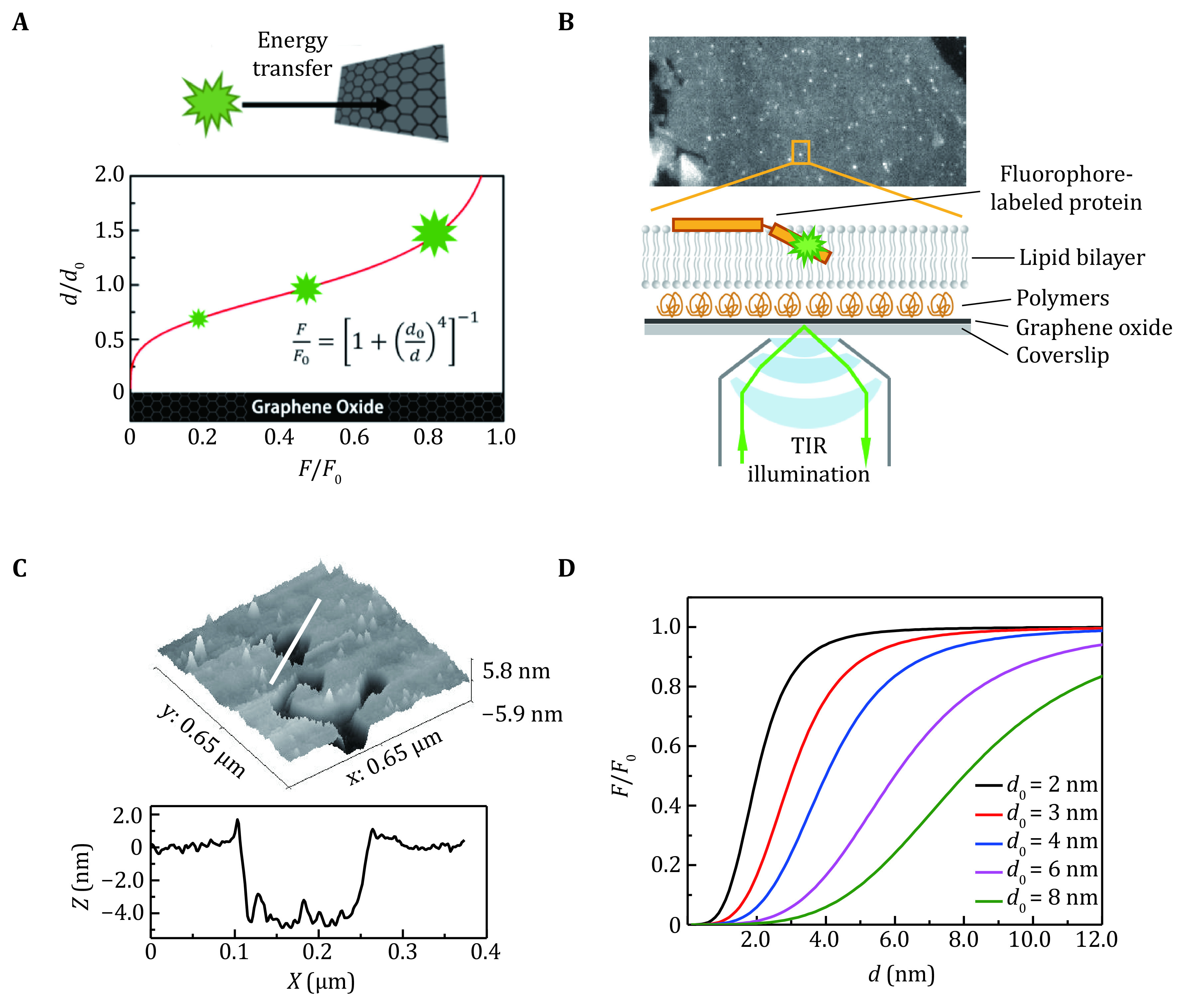
The principle and experimental sets of SIFA. **A** The fluorescence of a fluorophore changes rapidly with its distance to a graphene oxide layer. **B** The experimental setup of SIFA on TIRF microscopy. **C** The morphology (upper panel) and the profile of the thickness (lower panel) of a lipid bilayer composed of DOPC/DOPA. **D** The dependence of the relative intensity on distance in SIFA with different critical distances *d*_*0*_

SIFA makes use of the energy transfer from a fluorophore labeled on the membrane protein to a single layer of graphene oxide (Fig. 1A). The intensity of the fluorophore changes as it moves in the direction normal to the membrane laying on graphene oxide (Ghosh* et al.* 2021; Isbaner* et al.* 2018; Kim* et al.* 2010; Li* et al.* 2016). The spatial range of sensitivity of the energy transfer is near the membrane thickness, thus well suited for studying membrane protein dynamics. The intensity *F* of a fluorophore adjacent to the graphene oxide layer changes with the 4th power of the fluorophore-surface distance *d*, that is

1 $ \frac{F}{{F}_{0}}={\left[1+{\left(\frac{{d}_{0}}{d}\right)}^{4}\right]}^{-1}, $

where *F*_*0*_ is the intrinsic intensity of the fluorophore in the absence of the quenching surface, *d*_*0*_ refers to the critical distance (the point that relative intensity *F/F*_*0*_ is 0.5), which depends on the properties of the fluorophore and could be adjusted by the oxidation degree of the graphene oxide. Relative intensity *F/F*_*0*_ is monitored in the experiment and subsequently converted to the changes of the position *d*.

In the SIFA experiments, a coverslip is rendered hydrophilic with H_2_SO_4_/H_2_O_2_ solution or by an oxygen plasma cleaning procedure. Micrometer-sized monolayers graphene oxide is prepared following the modified Hummers method (Boychuk* et al.* 2019; Feicht* et al.* 2019) and deposited onto the coverslip with the Langmuir–Blodgett technique (Li* et al.* 2011; Mangadlao* et al.* 2015). Then the coverslips are reduced by heating in a vacuum to control the degree of oxidation. Subsequently, a layer of the supported lipid bilayer is formed on the graphene oxide through a vesicle-rupture method (Basu* et al.* 2020), or the solvent exchange method (Ferhan* et al.* 2019; Jackman and Cho 2020). Then membrane proteins with labeled fluorescence are integrated onto the lipid bilayer.

The fluorescence can be observed by various imaging techniques, but total internal reflection fluorescence (TIRF) microscopy (Fish 2009) is recommended in order to reduce the interference from the solution and to increase the signal-to-noise ratio during the single-molecule imaging (Fig. 1B). According to the AFM measurements, the thickness of a bilayer composed of DOPC and DOPA (7:3, molar ratio) is 4.5 ± 0.4 nm (Fig. 1C). Then the *d*_*0*_ value can be calibrated using the lipid bilayer with headgroup-labeled fluorescent lipid. Because the fluorescence of the labeled lipids which orient toward the graphene oxide is quenched completely, the fluorescence intensity or the lifetime of the fluorescent lipids at the upper surface could be measured to calibrate the *d*_*0*_ value according to Eq.1. In the SIFA experiments, apart from the intensity *F*, the value of the intrinsic fluorescence intensity *F*_*0*_ of the fluorophore should be measured at first under the same experimental condition because the distance *d* should be derived from the relative intensity *F/F*_*0*_.

An intensity-distance curve is shown in Fig. 1A. The relative intensity *F/F*_*0*_ increases rapidly as the fluorophore moves away from the graphene oxide layer. The distance in the range from 0.5*d*_*0*_ to 1.5*d*_*0*_ is the most sensitive region of the SIFA assay. The value of *d*_*0*_ could be adjusted by the reduction of the graphene oxide (Fig. 1D). In a series of researches (Li* et al.* 2016; Ma* et al.* 2018, 2019b; Xu* et al.* 2020), the *d*_*0*_ of the graphene oxide we used was 4.3 ± 0.5 nm, which means that the SIFA assay is sensitive to the position changes of a protein site located at positions from the upper leaflet of the supported lipid bilayer to 2–3 nm above the membrane surface if the lipid bilayer is directly deposited on the graphene oxide layer. That means the signal of the fluorophore at the deeper region of the membrane will be quite weak and lack response to the vertical movement. To solve this problem, a layer of polymers such as that of BSA or PEG can be deposited or modified on the graphene oxide prior to the supported lipid bilayer formation to form a “tethered supported lipid bilayer” (as shown in Fig. 1B). The polymers lift the membrane and push the deeper region of the membrane into the sensitive region of SIFA.

### The advantages and applications of SIFA

Benefited from the sensitivity of the energy transfer efficiency versus the off-surface distance, SIFA is a high-resolution method to probe membrane protein dynamics. It was already applied to characterize the trans-membrane intermediate states and the motions of antimicrobial peptides, as well as the oligomerization of a pro-apoptosis protein.

**Figure 2:**
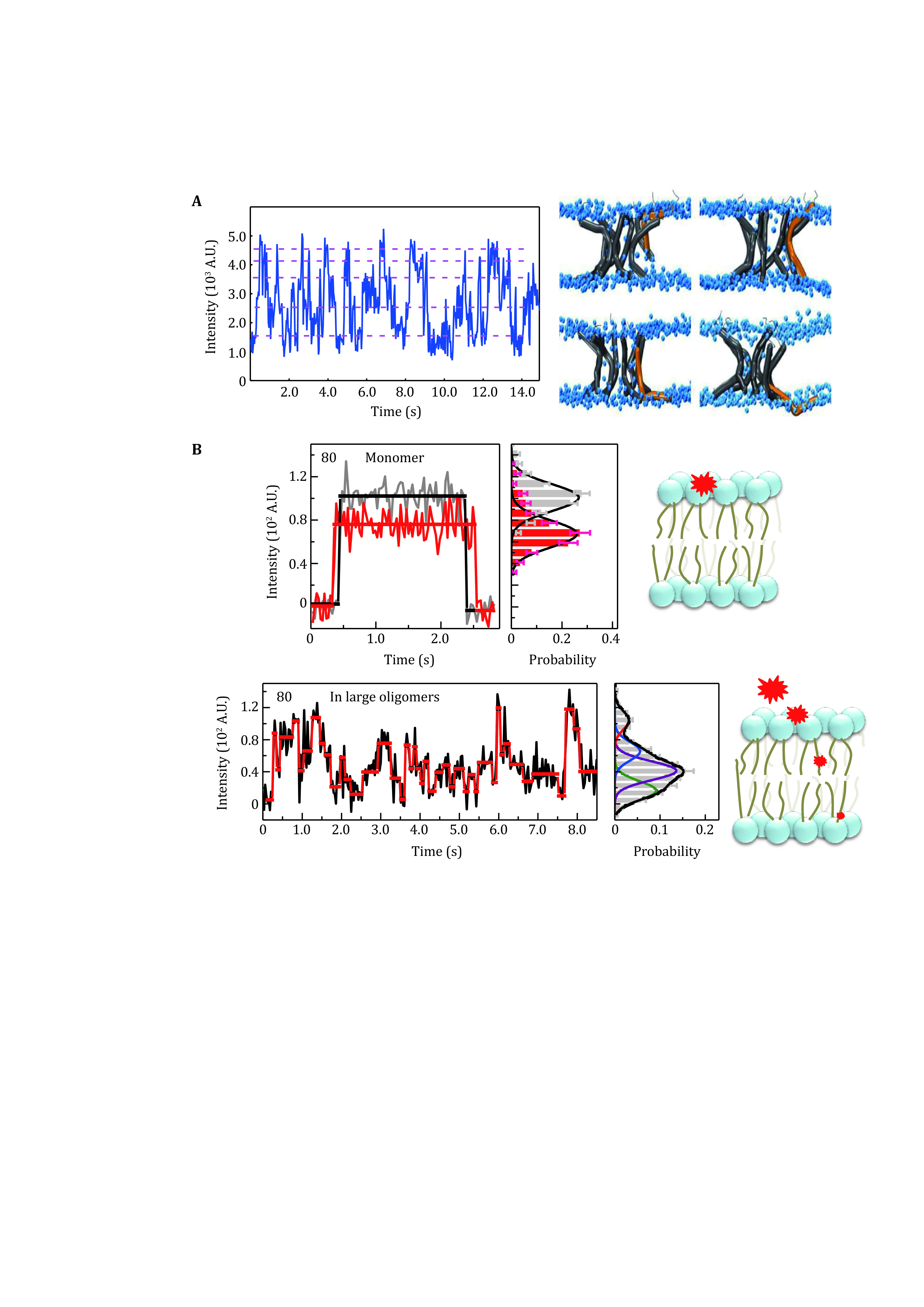
The applications of SIFA. **A** Dynamics of LL-37 N-terminal at a high peptide concentration (Li* et al.* 2016). The up-and-down motions are reflected by the fluorescence fluctuation in the trace (left panel), supporting the dynamic pore model (right panel). **B** The relatively static position of the residue 80 of a monomeric tBid (upper panel) and the upside-down movements of the same residue in large oligomers (lower panel) (Ma* et al.* 2019b)

The study on host-defense peptide LL-37 (Li* et al.* 2016) revealed transient insertion of a single peptide into the membrane and uncovered five relatively stable states of the peptide in the membrane at high surface density. The study supports a mode that the pores formed by LL-37 are dynamic (Fig. 2A). With fluorophore labeled on the phospholipids, the SIFA study on melittin (Xu* et al.* 2020) revealed that the peptide may induce flip-flops of single lipid molecules. The lipid headgroups showed a metastable state at the center of the lipid bilayer, suggesting that the melittin molecules form toroidal pores instead of barrel-like pores. The investigation of the pro-apoptosis protein tBid (Ma* et al.* 2019b) showed that tBid tends to stay at the membrane surface as monomers, but the protein may insert into the membrane when forming oligomers. Moreover, the penetration depths are different in the initial and mature stages of the self-assembly (Fig. 2B). These results showed that SIFA is powerful to study the membrane protein dynamics with the sub-nanometer vertical resolution (0.4–0.5 nm).

Another advantage of SIFA is that the micrometer-sized, solid-supported lipid bilayer is good for the observation of the lateral diffusion of molecules on the membrane. It means that the three-dimensional motions of the membrane-interacting proteins could be tracked. The study of tBid with SIFA observed the 3D motion of residue 166 in the central region of the protein on the membrane and identified the penetration depths of the site from different diffusion patterns (Ma* et al.* 2018). Notably, SIFA is compatible with multiple fluorescence imaging techniques. Combined with some new techniques aiming to locate single fluorophores below the diffraction limit (Gu* et al.* 2019), super-resolution 3D tracking of single membrane proteins would be possible. Prospectively, along with the localization and the tracking of the molecule in the x–y plane, SIFA can give information of the z-position, thus monitoring readily the inter-protein interactions, oligomer formation, or recruiting of the protein during the membrane phase separation.

## LIPOFRET: A LIPOSOME-BASED METHOD TO PROBE MEMBRANE PROTEIN DYNAMICS

### Basic principle and experiment set-up

**Figure 3:**
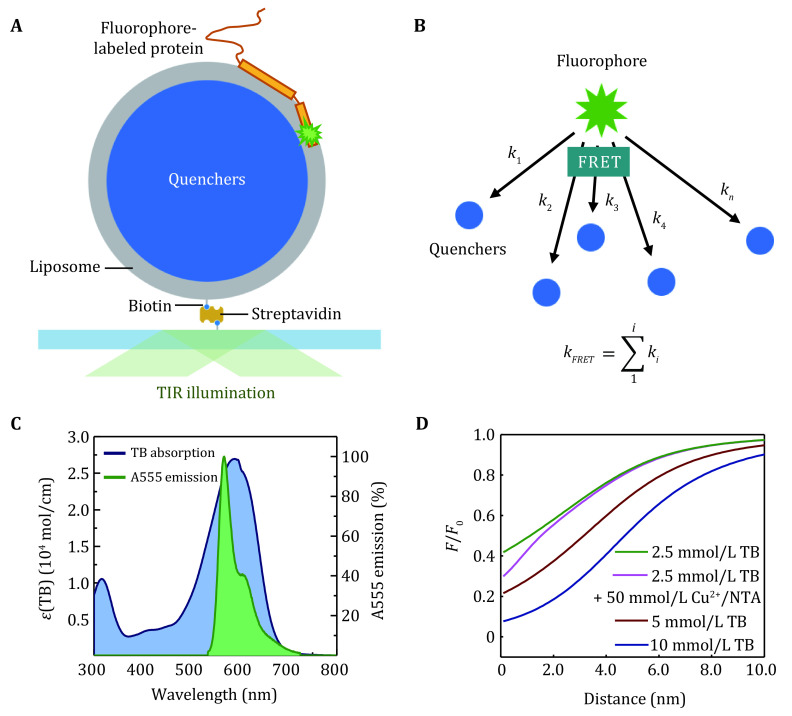
The principle of LipoFRET. **A** The scheme of LipoFRET. **B** FRET from one donor to multiple acceptors. **C** The absorption spectra of TB (blue) and the emission spectra of the donor Alexa Fluor 555 (green). **D** The dependence of the relative intensity on the distance to the inner surface of the liposome membrane in LipoFRET

Though SIFA can probe the membrane protein dynamics in real time, it still has some drawbacks. The solid substrate that supports the lipid bilayer may reduce the fluidity of the lipid bilayer, especially that of the lower leaflet in contact with the substrate (Schoch* et al.* 2018). Meanwhile, the planar lipid bilayer does not bear the curvature of the natural membrane of organelles, potentially limiting the capability to study some curvature-sensitive proteins (Jensen* et al.* 2011; Miller* et al.* 2015; Zeno* et al.* 2019). LipoFRET was developed based on liposomes, a bio-mimic system with surface curvature and unrestricted membrane fluidity. It is based on the principle of the FRET from one-donor to multiple acceptors (quenchers) encapsulated in unilamellar liposomes (Fig. 3A). Fluorophores on the membrane proteins with different penetration depths (or distance) in the liposome lipid bilayer show different intensities according to their energy transfer efficiency. For a single FRET pair, the energy transfer rate *k* is determined by

2 $ k=\frac{1}{{\tau }_{D}}{\left(\frac{{r}_{0}}{r}\right)}^{6}, $

where *τ*_*D*_ is the intrinsic lifetime of the donor, *r*_*0*_ is the critical distance (Fӧrster distance) which is determined by the spectra properties of the donor and acceptor, and *r* is the distance between the donor and the acceptor (Gennis and Cantor 1972).

In a one-donor and multiple-acceptor system, the transfer kinetics *k*_*t*_ can be written as the sum of pairwise rates (Frederix* et al.* 2002; Gennis and Cantor 1972; Lee * et al.* 2010) (Fig. 3B), that is:

3 $ {k}_{t}=\frac{1}{{\tau }_{D}}\sum _{i=1}^{N}{\left(\frac{{r}_{0i}}{{r}_{i}}\right)}^{6} , $

where *r*_*0i*_ and *r*_*i*_ are the Fӧrster distance and the spatial distance between the *i*th donor–acceptor pair, respectively. The energy transfer efficiency is then given by *E* =* k*_*t *_/(*τ*_*D*_^−1 ^*+ k*_*t*_), which is used to calculate the relative fluorescence of the donor, *F/F*_*0*_ = 1 –* E*, where *F*_*0*_ is the intrinsic fluorescence without the quenchers.

The choice of the quenchers for LipoFRET should follow the requirements: (1) There should be some overlap between the donor’s emission spectra and the quencher’s absorption spectra; (2) The quencher should have low quantum yield, or at least do not show strong emission around the detecting wavelength of the donor; (3) The quencher is not disruptive to the membrane of liposomes. Many dyes, including some organic dyes, or even some metal ions with proper absorption spectra, can serve as the quenchers. Trypan blue (TB), which is frequently used for live-cell counting in the dye-exclusion assay (Avelar-Freitas* et al.* 2014; Strober 2001), has been successfully used in current LipoFRET studies (Fig. 3C).

The Fӧrster distance between the donor and the quencher may be calculated from the spectrum (Vuojola* et al.* 2011), or deduced directly from the fluorescence of the fluorophore-labeled lipids in the liposomes. Because the distance between the quenchers is comparable to *r*_*0*_ in the LipoFRET experiments, the quencher solution cannot be treated as a continuous medium. A Monte Carlo simulation can be applied to calculate the relative intensity–distance relations (Fig. 3D). LipoFRET is also sensitive to the position changes of a fluorophore normal to the membrane. Although the membrane-quencher distance in LipoFRET cannot be adjusted as readily as in SIFA, raising the concentration of the quenchers can make* F/F*_*0*_ more sensitive to the region outside the membrane, while adding some short-range quenchers, such as Cu^2+^-nitrilotriacetic acid complex (Cu-NTA), can enhance the sensitivity in the deep region of the membrane.

LipoFRET could be implemented on different fluorescent imaging instruments. With the pseudo-TIRF illumination and EMCCD, single-molecule fluorescence can be acquired with a time resolution of tens of milliseconds. In the surface-immobilized observation, a coverslip is covalently coated with a layer of mPEG to avoid nonspecific adhesion. Biotin-PEG is used for the conjugation of liposomes (Diao* et al.* 2010). Unilamellar liposomes encapsulating the quenchers (usually prepared with the extrusion method to ensure the size homogeneity; Zhang 2017) are anchored on the surface through the specific biotin–streptavidin interaction. Fluorophore-labeled membrane proteins are reconstructed on or added to interact with the liposomes. Along with the control experiment measuring *F*_*0*_, which is the fluorophore intensity on liposomes without the quenchers, the relative intensity *F/F*_*0*_ is converted with the *F/F*_*0*_–distance curve.

### The application and features of LipoFRET

**Figure 4:**
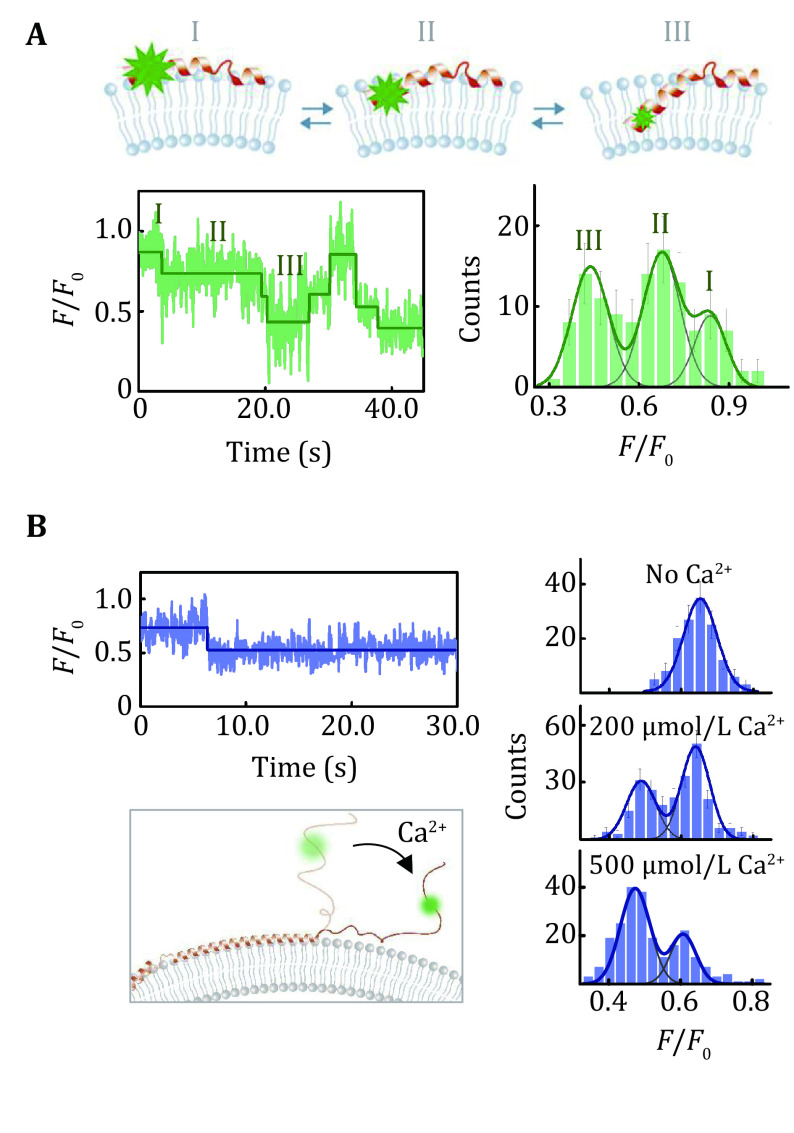
LipoFRET analysis of α-synuclein on the liposome membrane. **A** The spontaneous position changes of the N-terminal of α-synuclein (upper panel) and the corresponding intensity trace and distribution (lower panel) (Ma* et al.* 2019a). **B** Calcium-induced position changes of the acidic tail of α-synuclein (Ma* et al.* 2019a). The trace indicates the position changes of the C-terminal in the presence of Ca^2+^. The histograms showed the intensity distribution of the Alexa Fluor 555 labeled on residue 129 in the absence of Ca^2+^and in the presence of 200 μmol/L and 500 μmol/L Ca^2+^

Similar to SIFA, LipoFRET also provides high resolution insights into the position changes of membrane proteins in real-time. LipoFRET is based on artificial vesicles which are excellent model systems for studying the dynamics of membrane proteins. Generally, LipoFRET exhibits the flatter intensity–distance curve than does SIFA, thus it has a bit wider sensitive range but the slightly lower vertical resolution (about 0.6–0.7 nm). LipoFRET has been applied to study the membrane-interaction patterns of α-synuclein, the key player in the Parkinson’s disease (Ma* et al.* 2019a). LipoFRET distinguished the positions of the central NAC region and the acidic tail of the protein, and uncovered spontaneous dynamics of N-terminal among different depths in the membrane (Fig. 4A). Moreover, the addition of Ca^2+^ was found to switch the C-terminal tail to a new position closer to the membrane surface (Fig. 4B), showing that LipoFRET could be applied to study membrane protein conformational changes in protein–ligand interactions. A further study on the membrane binding of α-synuclein showed the regulation of protein concentration on its conformation (Ma* et al.* 2020). These studies demonstrated that LipoFRET is also a practical tool to study the membrane protein dynamics.

The advantage of LipoFRET also lies in its ability to change the surface curvature of the membrane. The curvature is adjustable through the control of the liposome diameter. Many membrane proteins are sensitive to the curvature as they interact with the membranes. α-Synuclein, for example, shows higher affinity with smaller-sized liposomes and may adopt different conformation compared to large liposomes (Caparotta* et al.* 2020; Middleton and Rhoades 2010). LipoFRET is compatible with different sizes of liposomes, hence the method is capable of investigating curvature-regulated membrane–protein interactions, or studying certain curvature-sensing membrane proteins in its optimal membrane environment.

In addition, because LipoFRET is based on liposomes, it has natural advantages in the research of physiological processes like synaptic vesicle recruiting, docking, releasing (Betz and Angleson 1998; Kweon* et al.* 2017; Saheki and De Camilli 2012), and GTPase-mediated membrane fusion or fission (Moon and Jun 2020; van der Bliek* et al.* 2013; Wang* et al.* 2019).

## QUEENFRET: FRET WITH QUENCHERS IN EXTRACELLULAR ENVIRONMENT

### The working principle of QueenFRET

**Figure 5:**
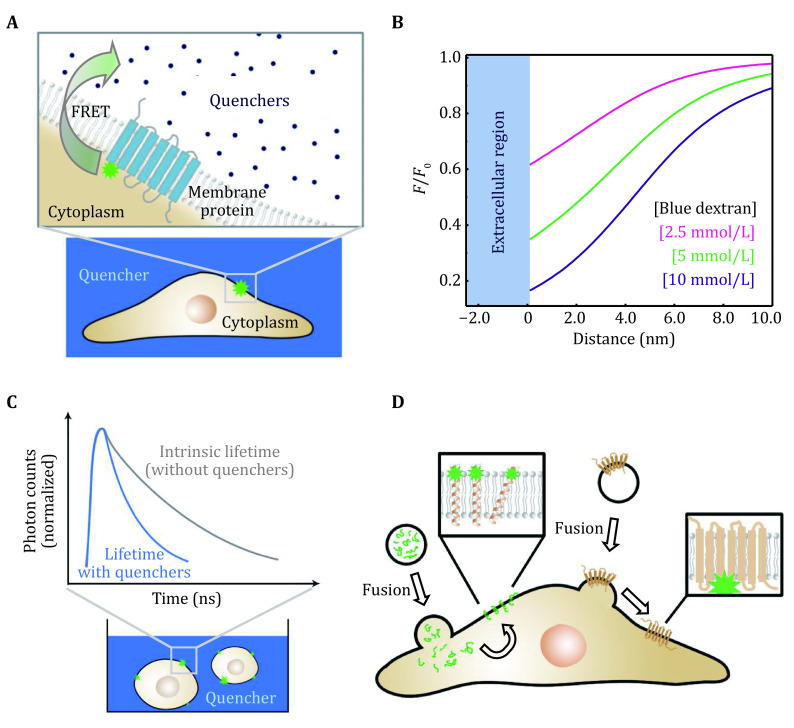
The principle and data acquisition of QueenFRET. **A** QueenFRET on live cell membranes. **B** Dependence of the relative intensity on the distance (in the direction inward the cell) to the outer surface of the cell membrane. **C** The scheme of the fluorescence lifetime measurements on live cells. The donors undergoing FRET have shortened fluorescence lifetimes (upper panel). **D** The incorporation of fluorophore-labeled peptides and proteins onto live-cell membranes

With well-designed approaches to make use of the energy transfer principle, SIFA and LipoFRET are able to image movements of macromolecules in model membranes. However, functions and dynamics of biomolecules in live-cell membranes may not be well represented by biomolecules in the artificial systems because of the lack of complex feedback networks or a crowding environment (Wang* et al.* 2019). Therefore, an *in-situ*, non-destructive method is necessary. QueenFRET, a real-time single-molecule method able to detect the motions of membrane protein on live cells was developed to meet this demand (Hou* et al.* 2021). QueenFRET also makes use of the FRET from one donor to multiple acceptors. The difference is that, in QueenFRET, the quenchers are added in the extracellular culture medium to attenuate the intensity of the fluorophores on proteins at cell membranes (Fig. 5A), resulting in a reversed intensity–distance curve (Fig. 5B) with respect to that of LipoFRET.

QueenFRET can also be carried out with pseudo-TIRF imaging, as long as the bottom of the cell is uniformly illuminated. It is also suitable to work along with the fluorescence lifetime imaging microscopy (FLIM). The lifetime of the donor decreases, following the same tendency as the intensity when FRET occurs (Wallrabe and Periasamy 2005), that is:

4 $ \frac{\tau }{{\tau }_{0}}=\frac{F}{{F}_{0}}=1-E , $

where *τ* and *F* are the measured lifetime and intensity of a donor fluorophore, *τ*_*0*_ and *F*_*0*_ refer to the intrinsic lifetime without quenchers, and *E* is the total FRET efficiency. Therefore, the principle of LipoFRET also applies to QueenFRET. The intensity increases as the fluorophore moves from the inner to the outer surface of the liposome in LipoFRET, whereas in QueenFRET, the intensity/lifetime decreases when the fluorophore moves from the cytoplasm out across the cell membrane.

As QueenFRET is a method designed for live-cell study, the quenchers must be biocompatible and non-toxic to cells. In addition, because of the complex chemical composition of bio-membranes, nonspecific adhesion of the quenchers onto the cell membranes should be avoided to reduce the influence on the quantitative interpretation of the data. In a previous study, blue dextran whose absorption maxima is around 600 nm was used as quenchers in our QueenFRET experiments for its favorable properties, such as neutrality, water solubility, and biocompatibility (Hou* et al.* 2021; Thompson* et al.* 1975).

The experimental procedure of QueenFRET is simple. For adherent cells, after the measurement of the fluorescence intensity or lifetime without the quenchers (*F*_*0*_ or *τ*_*0*_), blue dextran is dissolved in the cultural medium and added to the cultural dish for subsequent pseudo-TIRF or FLIM imaging of the cell bottom. The quencher percolates into the interspace between the cell and the cultural dish. In the case of floating cells, FLIM is preferred (Fig. 5C). Blue dextran can also be added and permeate into the microfluidic channels or even the interspace inside a cluster of cells. The labeling or incorporation of the membrane protein on/into live cells is a critical step in the QueenFRET measurements (Fig. 5D). One can reconstitute fluorophore-labeled membrane proteins onto vesicle membranes, or encapsulate water-soluble membrane-interacting proteins in vesicles, then incorporate them into the cell membranes through membrane-fusion (Tanaka and Schroit 1983). Another strategy is to label the proteins on the cell membranes *in situ* through some special tags, such as FlAsH or ReAsH (Machleidt* et al.* 2007; Moghaddam-Taaheri and Karlsson 2018). The Fӧrster distance *r*_*0*_ between the donor and blue dextran is measured from fluorophore-lipid containing liposome just as in the LipoFRET approach by fitting the curve given by the Monte Carlo simulation (Hou* et al.* 2021). Then the thickness of the cell membrane can be measured, and the position of the labeled site of the membrane protein normal to the membrane is derived with sub-nanometer precision.

### Applications and advances of QueenFRET

QueenFRET attenuates the fluorescence intensity of dyes located on the live cell membranes. This ensures the physiological significance of the results obtained. The application of QueenFRET on cellular membrane bound LL-37 had yielded similar results to that of SIFA, demonstrating the feasibility of the method (Hou* et al.* 2021).

**Figure 6:**
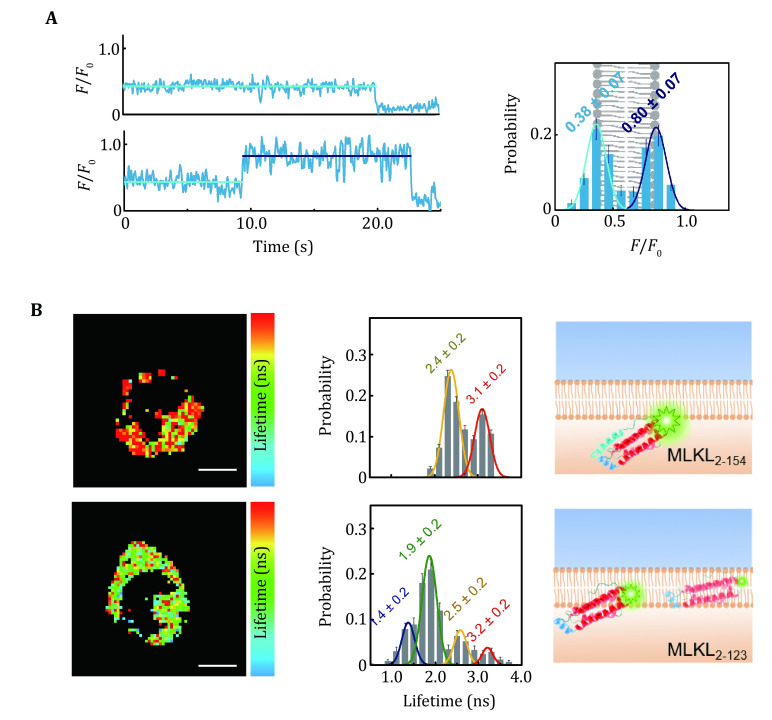
QueenFRET assay of interactions of biomolecules with live-cell plasma membranes. **A** QueenFRET measurement of flip-flops of lipid molecules on the cell membrane (Hou* et al.* 2021). The traces in the left panel show a lipid molecule whose headgroup points to the outer leaflet of the cell membrane (upper) and a lipid molecule that flips from the outer leaflet to the inner leaflet (lower). The histogram showed the intensity (position) distribution of the TAMRA-labeled lipids. **B** The locations of MLKL_2-154_ and MLKL_2-123_ in live cell membranes (Hou* et al.* 2021). The lifetime images (left panel) and the distributions (middle panel) clearly indicate the different in-membrane positions of MLKL_2-154_ and MLKL_2-123_

QueenFRET has been used to track the lipid flip-flop in live cell membranes at the single-molecule level (Hou* et al.* 2021), which is hard to be monitored with other methods (Fig. 6A). The maintenance of lipid asymmetry in the two leaflets of the plasma membranes can represent the activity of the flip-flop enzymes and reflect the cellular states. QueenFRET was also used to investigate proteins at the cytosolic side of the plasma membrane (Hou* et al.* 2021). The mixed lineage kinase domain-like protein (MLKL) (Fig. 6B), which is a key protein in necroptosis, was studied in THP-1 cells suspended in a microchip with lifetime imaging. QueenFRET had resolved the depth of the site S55 in short form (residues 2-123, termed as MLKL_2-123_) and long form (residues 2-154, MLKL_2-154_) in live cell membranes, showing that the S55 of MLKL_2-123_ inserts deeply in the hydrophobic core of the membrane, whereas that of MLKL_2-154_ tend to stay at the intracellular surface of the cell membrane. Compared to conventional fluorescence imaging methods, in which only tell whether a protein of interest is at the membrane or not, QueenFRET can further measure quantitatively the insertion depth or the distance relative to the membrane surface in real-time.

Prospectively, QueenFRET has the potential to be a powerful tool to study the response of the membrane proteins directly in the cell–cell interactions. The uncovering of the microscale-mechanism on bio-membranes would help understand the mechanism and regulation of some important physiological processes, such as cell clustering and adhesion, immunological recognition between T-cells and tumor cells, *etc*. QueenFRET is also being used to carry out the research on drug delivery across the cell membrane. Most importantly, as QueenFRET could be implemented with the fluorescence lifetime imaging, it is possible to work along with high-throughput cell-based screening methods to achieve microcosmic mechanism-based high-efficiency drug screening (Jager* et al.*^ 2003^).

## DATA COLLECTION AND PROCESSING

### Intensity measurements and interpretation

The intensity measurements in SIFA, LipoFRET and QueenFRET are commonly performed with fluorescence microscopy with total internal reflection (TIR) illumination to get high signal-to-noise ratios. Image collection with EMCCD would help probe the fluorescence signals with single-molecule sensitivity. In SIFA experiments, the lipid bilayer and the membrane proteins are within several nanometers to the solid surface. Hence the excitation laser with TIR illumination could be applied. However, in LipoFRET, the liposomes with tens or more than 100-nm diameters would occupy over the rapid exponential decay range of the TIR illumination (Fish 2009), leading to weaker excitation of the fluorophores on top of the liposome than at the bottom, therefore a broad intensity distribution and a reduced resolution. To get uniform excitation along the z-direction, it is better to have deep-penetration illumination in TIR, or to adopt the pseudo-TIR mode in which the incident angle is slightly lower than the critical angle (Kapanidis and Weiss 2002; Wang* et al.* 2017). For QueenFRET, although the basal membrane of the cells is usually observed, there may be a gap of several hundred nanometers so that the fluorophore should also be excited in the pseudo-TIR mode.

The fluorescence intensity of the donor fluorophore labeled on proteins should be stable enough so that the interference signal from the intrinsic fluctuation of the donor could be excluded. Signals of the fluorophores can be collected as image stacks for further process. The intensity information of the fluorescent spots representing single molecules may be extracted with Gaussian fitting or integration of the intensity profile, able to be achieved with many image-analyze tools. For complex traces containing multiple transition dynamics, several algorithms or tools exist to help search and analyze intermediate states (Bronson* et al.* 2009; McKinney* et al.* 2006; van de Meent* et al.* 2014). Finally, these states are converted to insertion-depths or distance to membrane surface through the intensity–distance curves.

### Lifetime measurement and processing in QueenFRET

Apart from the intensity measurement, FLIM with a time-correlated single photon counting system (TCSPC) is also commonly used in the QueenFRET experiments. Although lifetime imaging is usually not used as a single-molecule probing method, it visually displays the distribution of the studied macromolecules and specific dynamics over the cell membranes. Despite the fact that FLIM often takes tens of seconds to build a decay trace of lifetimes, the spatial and temporal resolutions could be balanced to extract the lifetime information of a small portion of the membrane proteins in short time windows. A pixel-by-pixel analysis can be applied to yield the lifetime distributions (Ghosh* et al.* 2019). Typically, >400 photons are required to ensure that the error is <10% when fitting a TCSPC curve to a single-exponential function (Ghosh* et al.* 2019). To achieve relative accurate fitting, pixels may be binned to shorten the time needed for data collection.

## SUMMARY AND PERSPECTIVE

Probing the structural dynamics of biomolecules in plasma membranes of live cells had ever been a very difficult task. By introducing the energy transfer from one fluorophore to a plane or a cloud of quenchers, SIFA, LipoFRET and QueenFRET have taken an explorative but powerful step to observe in real-time the motion of protein membranes with sub-nanometer resolution in the direction perpendicular to the membranes, from model systems like planar lipid bilayer and liposomes to live cells, each of which has its own advantages.

SIFA tracks position changes of membrane proteins on supported lipid bilayer with high resolution. The scale of the planar membrane also allows one to observe the lateral diffusion of the proteins. Combined with single-particle tracking (Calebiro and Sungkaworn 2018), the 3D motion of the labeled protein site can be tracked with high precision. Along with single-particle tracking and diffusion analysis, self-assembly processes such as dimerization of membrane receptors (Meng* et al.* 2014; Milligan* et al.* 2019) and oligomerization and pore formation of membrane-interacting peptides can be characterized simultaneously (Ma* et al.* 2019b), thus bridging the macroscopic behavior of the biomolecules with their microscale details. LipoFRET monitors protein dynamics on liposomes. Through the adjustment of liposome size, curvature-sensitive proteins such as α-synucleins are able to be studied in their favorable environment. The liposome system makes LipoFRET extremely suitable to study membrane proteins involved in physiological processes related to vesicles such as the proteins driving synaptic vesicle recycling, clustering, and releasing, and membrane fusion and fission, *etc*. QueenFRET can investigate membrane protein dynamics on living cell membranes. It studies macromolecular dynamics on membranes within a complete feedback network readily provided by the cell and does not need to establish *in-vitro* model systems, therefore ensuring the physiological relevance of the observed results. It is a promising technique to study the working mechanisms of membrane receptors during cell–cell interactions such as those in the immune response of T cells to tumor cells, and to accelerate the development of transmembrane drug delivery as well.

## Conflict of interest

Dongfei Ma, Wenqing Hou, Chenguang Yang, Shuxin Hu, Weijing Han and Ying Lu declare that they have no conflict of interest.
